# Inversion time optimization in postmortem 1.5 tesla FLAIR brain imaging: a pilot study

**DOI:** 10.1007/s00414-025-03571-6

**Published:** 2025-07-29

**Authors:** Christine Bruguier, V. Magnin, J-F Knebel, S. Grabherr, V. Dunet, P. Genet

**Affiliations:** 1 Department of Medical Radiology, Diagnostic and Interventional Radiology, Lausanne University Hospital and University of Lausanne, Rue du Bugnon 46, Lausanne, 1011 Switzerland; 2 Department of Medical Radiology, University Center of Legal Medicine of Lausanne- Geneva, Lausanne University Hospital and University of Lausanne, Vuillette 4, Lausanne 5, 1000 Switzerland; 3 University Center of Legal Medicine of Lausanne-Geneva, University Hospital of Geneva, Michel Servet 1, Geneva, 1205 Switzerland; 4 Faculty of Biology and Medicine of Lausanne, Lausanne, Switzerland; 5 Faculty of Medicine, University of Geneva, Geneva, Switzerland

**Keywords:** Postmortem MRI, 3D FLAIR sequence, Brain, Time inversion

## Abstract

**Introduction:**

Postmortem magnetic resonance imaging (PMMR) has gained importance during the last decade in forensic pathology. While many clinical radiology sequences are applicable for the evaluation of the brain, the 3D FLAIR sequence shows different contrast in postmortem cases compared to living patients. Two factors—the temperature and the interval between official declaration of the death and PMMR (DC-PMMR interval) are suspected to influence the optimal inversion time (TI) needed to achieve living patient-like image contrast.

This study aimed to investigate if our empirical approach had the same results as previous study.

**Materials and methods:**

3D FLAIR sequences with varying TI values (from 1660 ms to 900 ms, every 110 ms) were acquired. Two radiologists independently assessed the images, selecting the TI that produced the most patient-like contrast. Rectal temperature and the DC-PMMR interval were recorded, and Pearson correlation tests were conducted to evaluate interrelations between TI, temperature, and DC-PMMR interval. Interobserver reliability was assessed using PABAK.

**Result:**

Overall, 23 cases were analyzed. Rectal temperature ranged from 5.7 °C to 29.0 °C, and the DC-PMMR interval from 13.05 to 768 h. A moderate interobserver reliability (PABAK = 0.56) was observed. Significant correlations were observed between TI and both temperature (*r* = 0.70, *p* = 0.0014) and DC-PMMR interval (*r* = − 0.68, *p* < 0.0003).

**Conclusion:**

Our empirical approach trends the results of previous studies: Postmortem 3D FLAIR contrast is significantly affected by the temperature and the DC-PMMR interval, suggesting that TI should be adapted accordingly

## Introduction

Over the past decade, the use of radiological imaging in forensic medicine has increased considerably. Radiological techniques offer significant advantages in the evaluation of postmortem cases. For instance, multi-detector computed tomography (MDCT) provides comprehensive visualization of soft tissues, the skeleton, and internal organs without the need for invasive manipulation of the body prior to autopsy [1]. Furthermore, the administration of contrast medium via minimally invasive cannulation of the femoral vessels, as performed in multi-phase postmortem CT angiography (MPMCTA) [2, 3] enables detailed assessment of the vascular system. This imaging modality is, therefore, a valuable complementary and preparatory tool for autopsy procedures.

However, MDCT has certain limitations, particularly due to its low intrinsic soft tissue contrast, which reduces its effectiveness in evaluating solid organs and soft tissues such as the liver, musculature, and nervous system. This limitation is not observed with magnetic resonance imaging (MRI), which offers superior contrast resolution for these structures. While image contrast remains largely unchanged between clinical and postmortem images obtained with MDCT, postmortem magnetic resonance imaging (PMMR) often reveals notable differences in contrast, enhancing the visualization of anatomical and pathological details. Studies [4–11] indicate that changes in the relaxation times (T1 and T2) of soft tissues, organs, and bone marrow can impact the various contrast images encountered in PMMR. Nevertheless, PMMR has already been implemented in the routine daily practice of several forensic centers. Most of the PMMR protocols currently in use are based on clinical radiology practice and have been modified to closely align with established clinical standards.

Despite the possible changes in the contrast-image, informed radiologists can analyze these postmortem images for all sequences without encountering issues. One notable exception is the fluid-attenuated inversion recovery (FLAIR) sequence, which is known to perform poorly in cases involving low body temperature [12]. Quantitative assessments in postmortem imaging, such as brain or myocardial mapping and the Apparent Diffusion Coefficient (ADC) measurements, must be interpreted carefully, as postmortem changes can influence their reliability and diagnostic value [13–16].

In clinical radiology, FLAIR sequence, whether 2D or 3D, is commonly used in brain imaging to detect signs of multiple sclerosis, suspected child abuse (non-accidental head injury), inflammation or infection (e.g., encephalitis, herpetic infection), stroke, or acute bleeding. In this sequence, the signal from cerebrospinal fluid (CSF) is suppressed by applying a specific inversion time (TI) that corresponds to the longitudinal relaxation time of CSF [17–19]. Even in clinical settings, interpreting FLAIR images can be challenging, as factors such as dehydration can influence image quality [20].

**Fig. 1:**
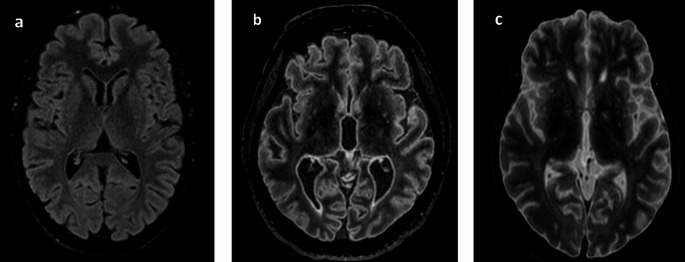
Axial reconstruction of a 3D sagittal cerebral FLAIR acquisition with a TI at 1660 ms: **a**) in the living at 36.8 °C; **b**) a corpse at 29.7 °C (Interval DC-PMMR: ~ 13 h); **c**) a corpse at 5.7 °C (Interval DC-PMMR: ~ 74 h)

**Fig. 2:**
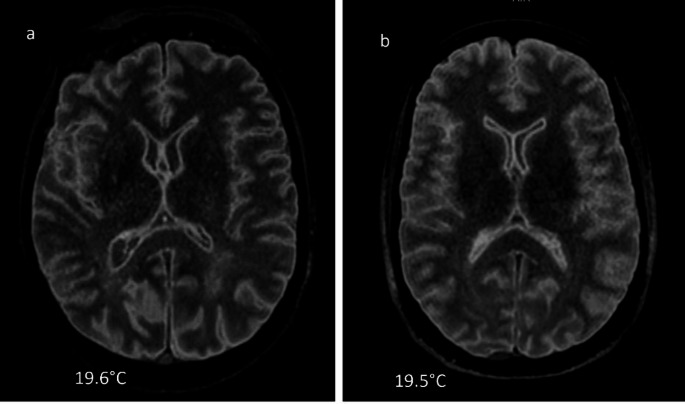
Axial reconstruction of a 3D sagittal cerebral FLAIR acquisition with a TI at 1660 ms: **a**) & **b**) two corpses at 19.5 °C with similar contrast image

In postmortem imaging, however, the contrast obtained with the 3D FLAIR sequence does not correspond to that of standard clinical 3D FLAIR images, making reliable interpretation difficult. Like other research teams, we have observed substantial contrast variability in this sequence across different postmortem cases. These discrepancies arise even when using the TI values recommended by the manufacturer for clinical use, for instance, 1660 ms on our imaging system (Figs. 1 and 2).

We also observed, fortuitously, that cases with similar body temperatures at the time of the MR examination exhibited comparable image contrast (Fig. 2).

According to the literature [5, 6, 21, 22], two variables appear to be primarily responsible for this phenomenon: the body temperature measured immediately prior to the PMMR examination, and the interval between the time of death certification (i.e., the official declaration of death by a physician) and the start of the PMMR scan (DC–PMMR interval).These variables can influence moreover a sequence parameter, the Inversion Time [12, 23–25].

Solutions were proposed by some authors to achieve image quality comparable to clinical images. These include, for example, extrapolation methods based on physical models quantifying the MRI signal [12] and the use of TI mapping derived from a 3D VIBE sequence, which differs from the conventional 3D FLAIR sequence [26].

However, we were unable to implement these approaches, primarily due to differences in sequence architecture (e.g., 2D vs. 3D acquisitions or variations in basic sequence parameters), as well as the complexity of certain algorithmic calculations, which proved impractical to perform in real time during PMMR acquisition [27].

Consequently, we opted for an empirical method compatible with the available tools at our disposal. We introduced rapid pre-sequences with empirically adjusted inversion times (TI) at regular, predefined intervals. Although these images were not suitable for diagnostic purposes, they allowed us to optimize image contrast and obtain interpretable results.

We chose this approach despite the availability of advanced tools, such as the “SyntheticMR, Linköping, Sweden”, which enables rapid post-processing reconstruction to generate images with various contrasts by adjusting parameters like TI, TR, and TE. Such tools, as described by some authors [28], can produce “synthetic” FLAIR images; however, we currently do not have access to these resources, as several other forensic institutes.

By adapting the inversion time (TI) using our empirical method, we achieved a significant improvement in the image contrast of the 3D FLAIR sequence. However, the choice of the time for TI modification was influenced by the individual radiographer’s judgment and experience. To ensure consistent diagnostic image quality, it was necessary to address this variability. Given the empirical nature of our approach, we first sought to verify whether our data exhibited similar trends to those reported in previous studies before proposing a reproducible solution.

The aim of this study was to assess whether the relationship between TI in the 3D FLAIR sequence and body temperature observed in our data corresponded to that reported in the literature. Additionally, we evaluated the correlation between TI and the interval from death certification to the start of PMMR (DC–PMMR interval), as described in prior studies.

## Materials and methods

### Cohort

This retrospective pilot study included all cases that underwent brain PMMR at our forensic center in 2018. Inclusion criteria were: availability of brain PMMR images with varying inversion times, recorded rectal temperature before MRI, and known post-mortem interval. Characteristics of the cases (sex and age) were recorded. We included brain PMMR images where the pre-sequences showed different inversion times (TI).

Our exclusion criteria were, a not recorded rectal body temperature and/or DC-PMMR interval, or a refusal of consent for the use of data for further research purposes.

### PMMR acquisition

Before acquiring the 3D FLAIR sequence used for diagnostic interpretation, we performed seven rapid, modified 3D FLAIR sequences in sagittal plane. The duration for all these specific acquisitions was 9 min. We used a 1,5T MR scanner (Philips, Netherlands) and we varied the TI every 110 ms, from 1660 ms (original clinical TI for a 1.5T in our MRI) to 900 ms, with a TR/TE of 4800/300 ms. We used a slice thickness of 1.15 mm, in-plane resolution of 1.15 × 1.15 mm^2^ and a secondary reconstruction in axial plane with a slice thickness of 3 mm. All the examinations were performed using a 16-channel head coil.

### Images evaluation

**Fig. 3:**
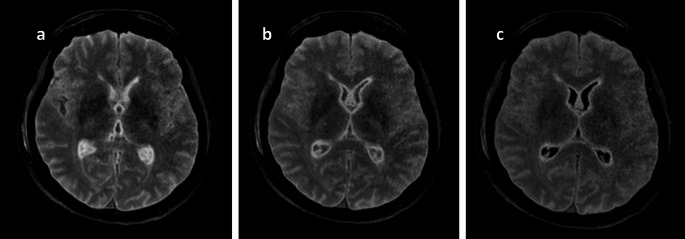
Example of FLAIR axial reconstructions of the brain for a case at 16,1 °C: **a**) TI 1660 ms (original); **b**: TI 1440 ms; **c**: TI 1220 ms, in which the image **c**) was considered as a living-patient like contrast, with the CSF signal suppressed (appearing black on the image)

All cases included in the study were coded form case 1 to case 24. Secondary axial plane reconstructions with a thickness of 3 mm were generated from the sagittal images of the 3D FLAIR sequence covering the brain. These axial images were independently and blindly evaluated by two experienced radiologists: a senior neuroradiologist with over 10 years of experience and a senior general radiologist with more than 10 years of experience, including 5 years in postmortem imaging. Using Advantage Windows workstations (GE Healthcare, Milwaukee, USA) under identical reading conditions, they selected the TI value corresponding in their opinion to the complete suppression of the CSF signal, in order to achieve a FLAIR contrast similar to that of a standard clinical case (Fig. 3). The TI values were then recorded in an Excel table.

### Statistical analyses

We performed statistical analyses with the Stata 16.0 software^®^. The Prevalence-Adjusted and Bias Adjusted Kappa (PABAK) was used to assess the agreement between the two observers. The values were interpreted as followed: 0 was considered poor; 0.01–0.20 as slight; 0.21–0.40 as fair; 0.41–0.60 as moderate; 0.61–0.80 as substantial; and 0.81-1.00 as almost perfect.

Pearson’s correlations were performed between the mean TI selected by the two observers and the rectal temperature measured before the PMMR; the mean TI and the DC-PMMR interval. We also performed a Pearson’s correlation test between body temperature and the DC-PMMR interval. The correlation values were considered: none if r<|0.1|; weak if r was between |0.1| and |0.30|; moderate if r was between |0.31| and |0.50|; substantial if r was between |0.51| and |0.70|; strong if r was between |0.71| and |0.99|; and exact if r=|1|. Finally, correlation tests were considered significant with a p-value < 0.05. These Pearson’s tests were followed by a linear regression.

## Results

**Fig. 4:**
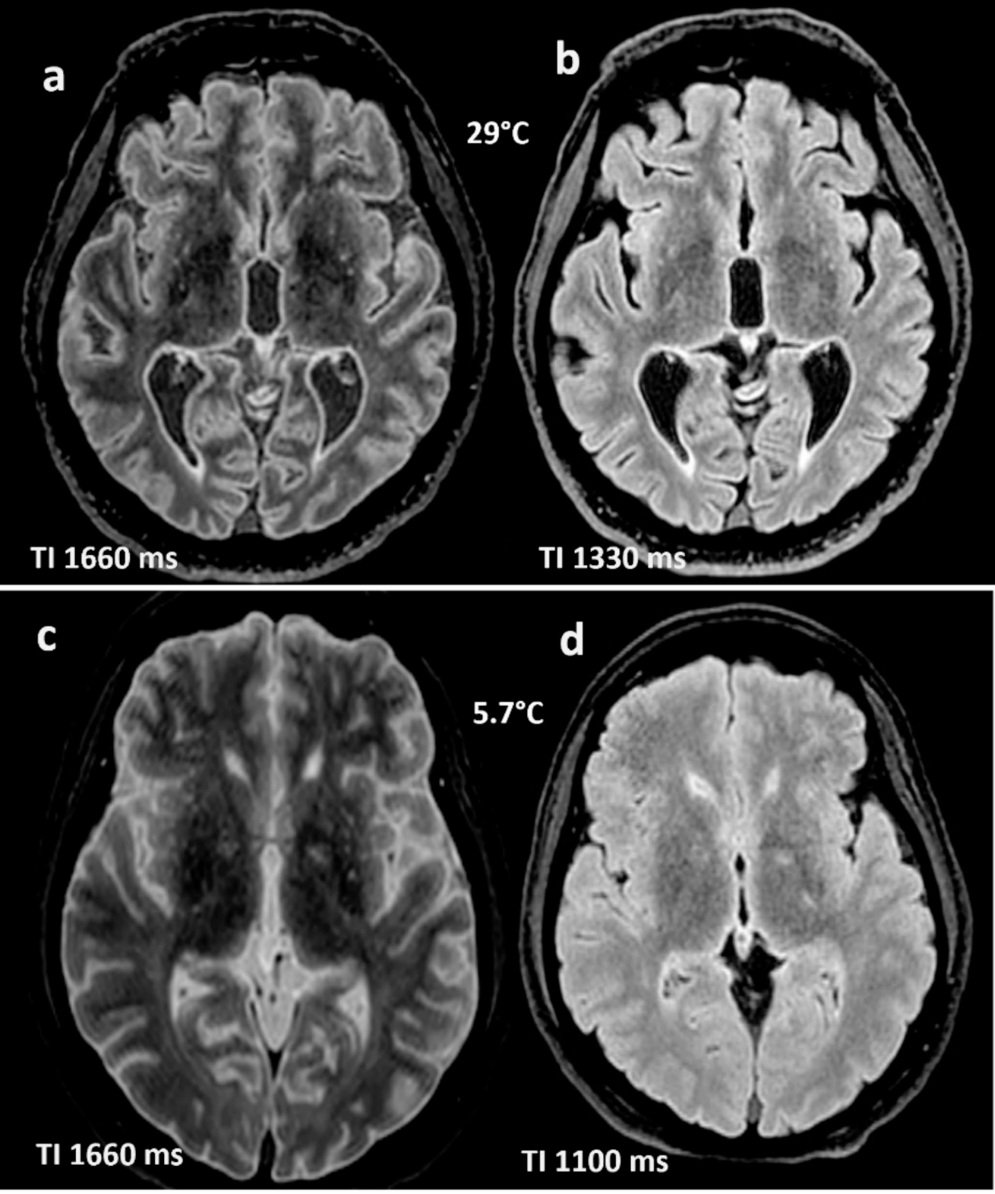
Example of axial FLAIR reconstructions of the brain with different TI. a) and b): same case at 29 °C; **a**: TI original 1660 ms and optimal **b**: TI 1330 ms; c) and d): same case at 5.7 °C; **c**: TI original 1660 ms and optimal **d**: TI 1100 ms

Our final sample size was *n* = 23, consisting of 5 females and 18 males with ages ranging from 17 to 94 years (median 54 years). The rectal temperature measured before the start of the PMMR ranged from 5.7 °C to 29.7 °C (median 17 °C). The DC-PMMR, corresponding to 12.2 h to 74.2 h (median 30 h). The optimal mean TI value ranged from 1100 ms to 1330 ms (median 1220 ms), Fig. 4.

Interobserver reliability was moderate, PABAK [95% CI] = 0.56 [0.28–0.84].

**Fig. 5:**
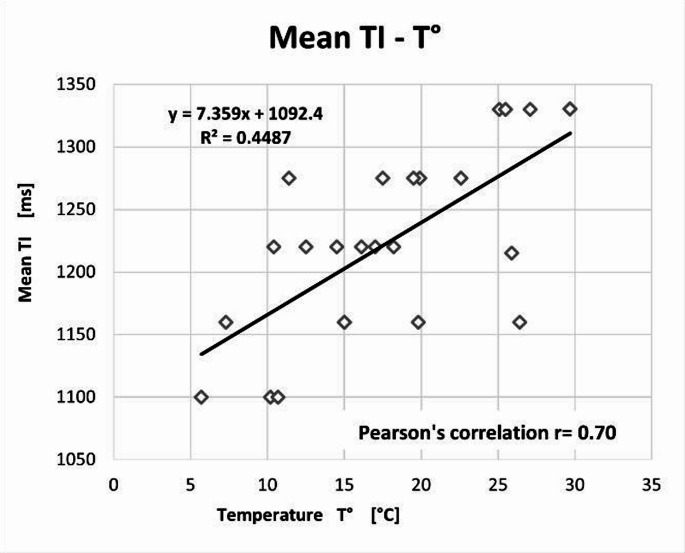
Pearson’s correlation between the mean TI and the body temperature *r* = 0.70, *p* < 0.0014

**Fig. 6:**
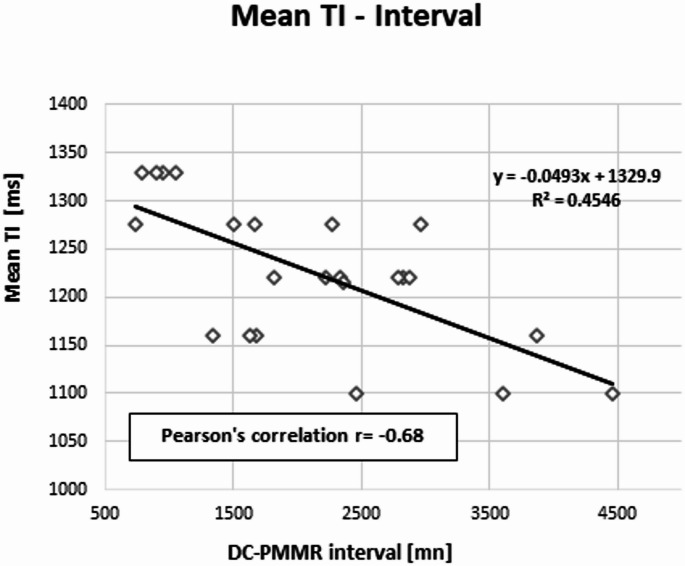
Pearson’s correlation between the mean TI and the DCD-PMMR interval, *r*= − 0.68, *p* < 0.0003

**Fig. 7:**
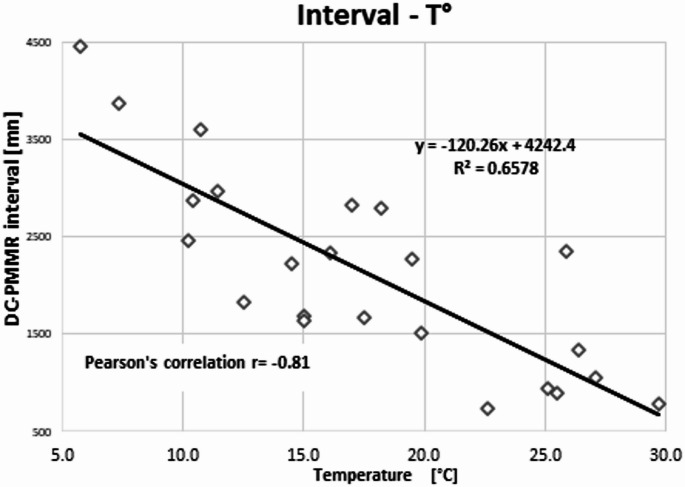
Pearson’s correlation between the body temperature and the DC-PMMR interval, *r*= − 081, *p* < 0.0001

The correlation between temperature and TI was *r* = 0.70 with a p-value of 0.0014 (Fig. 5); the correlation between TI and the interval between the DC- PMMR interval was *r* = − 0.68 with a p-value < 0.0003 (Fig. 6); and the correlation between the two covariates was *r* = − 0.81 with a p-value < 0.0001 (Fig. 7).

## Discussion

The purpose of our study was to verify whether our empirical approach followed the same trend regarding the relationship between the inversion time (TI) of the 3D FLAIR sequence and body temperature. We also evaluated the correlation between TI and the interval from death certification to the beginning of PMMR (DC–PMMR interval).

Our primary observation is that lower body temperature requires shorter TI to achieve an image contrast like that of living patients (Fig. 5). This finding was first described more than a decade ago by Tofts et al. [12]. Understanding this relationship is critical, as the 3D FLAIR sequence is an essential tool for assessing the central nervous system in PMMR, particularly when detecting pathologies such as acute bleeding or stroke.

As noted in the introduction, we were unable to implement previously proposed methods for overcoming FLAIR-related issues in postmortem imaging due to several factors: complex algorithms, differences in sequence architecture (2D vs. 3D), use of MRI systems from different manufacturers, and unavailability of specific sequences (e.g., 3D VIBE or T1 mapping) on our machine [12, 26–28].

Despite using an empirical approach, our results confirmed the influence of body temperature on the TI of the 3D FLAIR sequence. We observed a substantial linear correlation (*r* = 0.70) between these variables, with TI decreasing as body temperature declines. Abe et al. [26] reported similar results in a sample of 28 cases, using a T1 mapping approach of cerebrospinal fluid, which is complex and time-consuming. Similarly, Berger et al. [27], who investigated the most reliable anatomical site for temperature measurement (rectal, forehead, or intracerebral) to adjust TI, found a Pearson correlation of *r* = 0.79 between TI and rectal temperature. However, their method involves complex mathematical calculations and brain segmentation, making it unsuitable for rapid case management.

Regarding the relationship between TI and the DC–PMMR interval, we observed a substantial negative linear correlation (*r* = − 0.68), with TI decreasing over time. We also found that body temperature and the DC–PMMR interval are collinear, which is expected in forensic practice [29, 30]. Given that the DC–PMMR interval is influenced by several uncontrolled factors (such as body storage conditions and most of the time the ignorance of the exact time of death), this collinearity is noteworthy. Practically, this means that the measurable variable, namely the body temperature, can be used alone to adjust TI. This insight could enable the development of a mathematical formula for TI adjustment even when the DC–PMMR interval is unknown. However, given our limited sample size (*n* = 23), it remains premature to establish a robust formula at this stage.

The moderate interobserver concordance (PABAK of 0.56), despite identical image evaluation conditions, suggests that the 110 ms interval might be too large, causing a significant gap between two contrast-images, forcing radiologists to choose a TI far from the desired patient-like contrast-image. Nonetheless, the moderate PABAK supports one of our initial points, namely the difficulty in selecting the best TI during the exam, indicating the need for a reproductive solution.

## Limitations

The main limitation of this study is the relatively small sample size, which precludes the development and validation of a reproductible solution for TI adjustment. Second, images were analyzed by two board-certified radiologists, which overall could limit the variability of inter-rater evaluation and may influence the reported PABAK values. Third, this pilot study focused exclusively on evaluating image contrast and did not assess the impact of varying inversion times on the signal-to-noise ratio (SNR) of the images. Future investigations should address this aspect and explore whether other acquisition parameters require adjustment to optimize overall image quality in postmortem 3D FLAIR imaging.

## Conclusion

Our study confirmed trends reported in previous researches, demonstrating that both body temperature and the interval between death certification and the start of PMMR influence the inversion time (TI) of the 3D FLAIR sequence. These findings validate the usability of our empirical approach, an accessible method that does not require being an MRI specialist. With further refinement, this method could lead to a reproducible and practical solution for determining the optimal TI in postmortem imaging.

## Abbreviations

DC: Death certification time
FLAIR: Fluid Attenuation Inversion Recovery
MRI: Magnetic Resonance Imaging
pm: postmortem
PMMR: Postmortem Magnetic Resonance
PABAK: Prevalence-Adjusted and Bias-Adjusted Kappa
TI: Inversion Time

## References

[1] Chevallier C et al (2013) Postmortem computed tomography angiography vs. conventional autopsy: advantages and inconveniences of each method. Int J Legal Med 127(5):981–989 23292183 10.1007/s00414-012-0814-3

[2] Grabherr S et al (2011) Multi-phase post-mortem CT angiography: development of a standardized protocol. Int J Legal Med 125(6):791–802 21057803 10.1007/s00414-010-0526-5

[3] Grabherr S et al (2018) Postmortem CT angiography compared with autopsy: a forensic multicenter study. Radiology 288(1):270–276 29714682 10.1148/radiol.2018170559 PMC6027995

[4] Petren-Mallmin M et al (1993) The effect of temperature on MR relaxation times and signal intensities for human tissues. Magma 1:176–184

[5] Ruder TD et al (2012) The influence of body temperature on image contrast in post mortem MRI. Eur J Radiol 81(6):1366–1370 21458188 10.1016/j.ejrad.2011.02.062

[6] Zech WD et al (2015) Temperature dependence of postmortem MR quantification for soft tissue discrimination. Eur Radiol 25(8):2381–2389 25636417 10.1007/s00330-015-3588-4

[7] Baron P et al (2015) T1 and T2 temperature dependence of female human breast adipose tissue at 1.5 T: groundwork for monitoring thermal therapies in the breast. NMR Biomed 28(11):1463–1470 26403166 10.1002/nbm.3410

[8] Tashiro K et al (2015) Cerebral relaxation times from postmortem MR imaging of adults. Magn Reson Med Sci 14(1):51–56 25500777 10.2463/mrms.2013-0126

[9] Zech WD et al (2015) Postmortem quantitative 1.5-T MRI for the differentiation and characterization of serous fluids, blood, CSF, and putrefied CSF. Int J Legal Med 129(5):1127–1136 26162597 10.1007/s00414-015-1218-y

[10] Webb B et al (2017) Temperature dependence of viscosity, relaxation times (T(1), T(2)) and simulated contrast for potential perfusates in post-mortem MR angiography (PMMRA). Int J Legal Med 131(3):739–749 27900508 10.1007/s00414-016-1482-5 PMC5388705

[11] Adolphi NL (2016) An equation-free introduction to post-mortem MR image contrast and pulse sequence optimization. J Forensic Radiol Imaging 4:27–34

[12] Tofts PS et al (2008) Imaging cadavers: cold FLAIR and noninvasive brain thermometry using CSF diffusion. Magn Reson Med 59(1):190–195 18058937 10.1002/mrm.21456 PMC2478723

[13] Birkl C et al (2014) Temperature-induced changes of magnetic resonance relaxation times in the human brain: a postmortem study. Magn Reson Med 71(4):1575–1580 23716457 10.1002/mrm.24799

[14] Schmidt TM et al (2012) DWI of the brain: postmortal DWI of the brain in comparison with in vivo data. Forensic Sci Int 220(1–3):180–183 22445270 10.1016/j.forsciint.2012.02.022

[15] Alderliesten T et al (2015) Therapeutic hypothermia modifies perinatal asphyxia-induced changes of the corpus callosum and outcome in neonates. PLoS One 10(4):e0123230 25923113 10.1371/journal.pone.0123230 PMC4414268

[16] Youn CS et al (2015) Repeated diffusion weighted imaging in comatose cardiac arrest patients with therapeutic hypothermia. Resuscitation 96:1–8 26206595 10.1016/j.resuscitation.2015.06.029

[17] De Coene B et al (1992) MR of the brain using fluid-attenuated inversion recovery (FLAIR) pulse sequences. AJNR Am J Neuroradiol 13(6):1555–1564 1332459 PMC8332405

[18] Hajnal JV et al (1992) Use of fluid attenuated inversion recovery (FLAIR) pulse sequences in MRI of the brain. J Comput Assist Tomogr 16(6):841–844 1430427 10.1097/00004728-199211000-00001

[19] Blüml S et al (1993) Spin-lattice relaxation time measurement by means of a turboflash technique. Magn Reson Med 30(3):289–295 8412599 10.1002/mrm.1910300304

[20] Manuel BJ (2021) Evaluation des altérations cérébrales liées à l’anorexie mentale par des techniques avancées IRM. Université de Genève. p. 58

[21] Jackowski C et al (2006) Postmortem imaging of blood and its characteristics using MSCT and MRI. Int J Legal Med 120(4):233–240 16328426 10.1007/s00414-005-0023-4

[22] Kobayashi T et al (2010) Characteristic signal intensity changes on postmortem magnetic resonance imaging of the brain. Jpn J Radiol 28(1):8–14 20112087 10.1007/s11604-009-0373-9

[23] Bolen G et al (2010) Magnetic resonance signal changes during time in equine limbs refrigerated at 4 degrees C. Veterinary radiology & ultrasound, 51:19–24. 1 10.1111/j.1740-8261.2009.01615.x 20166388

[24] Kobayashi T et al (2010) Postmortem magnetic resonance imaging dealing with low temperature objects. Magn Reson Med Sci 9(3):101–108 20885082 10.2463/mrms.9.101

[25] Kobayashi T et al (2014) Optimization of inversion time for postmortem short-tau inversion recovery (STIR) MR imaging. Magn Reson Med Sci 13(2):67–72 24769635 10.2463/mrms.2013-0046

[26] Abe K et al (2015) Optimization of inversion time for postmortem fluid-attenuated inversion recovery (FLAIR) MR imaging at 1.5T: temperature-based suppression of cerebrospinal fluid. Magn Reson Med Sci 14(4):251–255 25833274 10.2463/mrms.2014-0086

[27] Berger C et al (2022) Technical note: quantitative optimization of the FLAIR sequence in post mortem magnetic resonance imaging. Forensic Sci Int 341:111494 36242925 10.1016/j.forsciint.2022.111494

[28] Okawa R et al (2023) Optimization of the fluid-attenuated inversion recovery (FLAIR) imaging for use in autopsy imaging of the brain region using synthetic MRI. Technol Health Care 31(2):661–674 36093648 10.3233/THC-220230

[29] Henssge C (1988) Death time estimation in case work. I. The rectal temperature time of death nomogram. Forensic Sci Int 38(3–4):209–236 3192144 10.1016/0379-0738(88)90168-5

[30] Henssge C, Madea B (2004) Estimation of the time since death in the early post-mortem period. Forensic Sci Int 144(2):167–175 15364387 10.1016/j.forsciint.2004.04.051

